# Health and social outcomes of HIV‐vulnerable and HIV‐positive pregnant and post‐partum adolescents and infants enrolled in a home visiting team programme in Kenya

**DOI:** 10.1111/tmi.13568

**Published:** 2021-03-25

**Authors:** Marcy Levy, Malia Duffy, Jennifer Pearson, Job Akuno, Samuel Oduong, Aida Yemaneberhan, Alexandra Coombs, Nicole Davis, Isabella Yonga, Rose Kerubo Mokaya

**Affiliations:** ^1^ International Division John Snow, Inc. Boston MA USA; ^2^ Elizabeth Glaser Pediatric AIDS Foundation Nairobi Kenya; ^3^ Elizabeth Glaser Pediatric AIDS Foundation Washington DC USA; ^4^ United States Agency for International Development Kenya and East Africa Village Market Nairobi Kenya

**Keywords:** pregnancy, post‐partum, HIV, adolescents, infants, home visiting

## Abstract

**Objectives:**

HIV‐positive and HIV‐vulnerable pregnant adolescent girls and adolescent mothers face significant barriers and vulnerabilities. Infants born to adolescent mothers are also more likely to die and be exposed to life‐threatening conditions. This paper presents findings from an evaluation of a programme that used a home visitation model and offered a case‐management, team‐focused approach to increase family and community supportiveness to enhance health and social service uptake among pregnant adolescent girls and adolescent mothers in Kenya.

**Methods:**

The study used a quasi‐experimental design with before and after comparisons among a non‐randomised population to examine the effectiveness of bi‐monthly household visits to 384 enrolled pregnant adolescent girls, adolescent mothers (ages 10–19) and their infants (0–24 months) between March 2018 and February 2019 in three counties in Kenya.

**Results:**

During the programme, household support increased from 57% to 85%, while 100% of eligible participants were on ART and virally suppressed (total of 20 adolescents). Nearly all pregnant adolescent girls (94%) delivered under skilled care vs. 78% of those who were post‐partum at the time of enrolment (*P* < 0.001); 100% of infants (total of 17 infants) had an up‐to‐date PCR test with no seroconversions. Uptake of modern family planning increased from 39% at baseline to 64% at end line (*P* < 0.001). The referral rate declined from 84% to 78% from baseline to end line with low uptake of referrals for mental health services (17.3%).

**Conclusions:**

A team‐focused approach of home visits to HIV‐vulnerable and HIV‐positive pregnant and post‐partum adolescent girls and their infants combined with efforts to reduce stigma and increase supportiveness of households and the community can help address critical socio‐cultural and behavioural barriers to accessing and using health and social services.

## Introduction

One in five adolescent girls in sub‐Saharan Africa becomes pregnant by age 18 – a rate that will result in an estimated 16.4 million adolescent mothers by 2030 [1, 2, 3]. Complications during pregnancy and childbirth and reduced access to maternal health services significantly contribute to adolescent maternal morbidity and mortality [4, 5, 6]. Babies born to adolescent mothers are 50% more likely to die at birth or in their first week of life than those born to young women in their twenties [5, 7].

Adolescent girls who are pregnant or raising a child face a host of barriers, including stigma and discrimination from household and community members; maltreatment by school administrators, healthcare providers and/or the police; trauma if they became pregnant as a result of sexual violence; and economic insecurity [5, 8, 9, 10]. They may need to drop out of school and/or face challenges in returning to school or work while caring for the new baby [11, 12].

In high HIV prevalence settings, adolescent girls face concurrent risks of HIV and pregnancy, including vertical HIV transmission to their infants. Studies in sub‐Saharan Africa show that compared with older women, adolescent girls are less aware of their HIV status, have lower antiretroviral treatment (ART) uptake, higher loss to follow‐up from prevention of mother‐to‐child transmission (PMTCT) programmes, reduced uptake of early infant diagnosis and increased rates of mother‐to‐child transmission of HIV [13, 14, 15, 16, 17, 18, 19]. In Kenya, where there are an estimated 1.6 million HIV‐positive individuals (with prevalence rate among adults 15–49 years being 4.5%), adolescent girls 15–24 years make up one‐third of all new adult HIV infections, and 26% give birth by age 18 [3, 20, 21, 22, 23].

In recent years, home visiting models have been adapted to support pregnant and post‐partum women and their infants to access and use HIV services. However, implementation varies widely, making it difficult to demonstrate programmatic benefits, especially through the PMTCT cascade [24]. Additionally, there are a dearth of home visiting programmes designed specifically for pregnant adolescents girls, adolescent mothers and their infants, despite the significant vulnerabilities that they face [25].

In order to address the vulnerabilities of pregnant adolescent girls, adolescent mothers and their infants, government and non‐governmental partners in Kenya designed a programme that involved a home visiting team (HVT) model aimed at improving their HIV and other health and social outcomes. The programme, known as The Strengthening High Impact Interventions for an AIDS‐free Generation (AIDSFree) Jielimishe Uzazi Na Afya (JUA) in Kenya, was a critical step towards determining the potential contribution of home visiting programmes to reduce health and social vulnerabilities among HIV‐positive and HIV‐vulnerable pregnant and post‐partum adolescent girls and their children in sub‐Saharan Africa. This paper presents findings of an evaluation of the programme to determine its impact on health and social outcomes of pregnant adolescent girls, adolescent mothers and their infants.

## Methods

### Study design

We used a quasi‐experimental study design with before and after comparisons among the same group of non‐randomised participants at both time points. The programme enrolled 384 pregnant adolescent girls and adolescent mothers (ages 10–19 years) from March 2018 to February 2019 in two wards in Homa Bay County (Kendu Bay Town and Wangchieng), three wards in Kisumu County (Kajulu, Kolwa East and Miwani) and four wards in Nairobi County (Karura, Kangemi, Kabiru and Gatina). We selected these counties based upon the high prevalence of HIV and pregnancy among adolescent girls. We collected primary data from adolescent girls, their families/caregivers and partners, which were supplemented with secondary data as follows: (i) programme data collected during home visits; (ii) routine service delivery data extracted from health facility registers; and (iii) routine service delivery data from community‐based organisations.

### Home visiting programme intervention description

The intervention involved different activities implemented by three cadres of community resource persons. The first cadre involved *mentors* who were young mothers aged 19–30 years trained to mentor adolescent girls to access and remain in services, care for themselves and their children. The second cadre included *household facilitators* (male and female) who mentored and educated other community members in the adolescents’ lives – caregivers, partners, school administrators and health facility management – to address barriers to care, reduce stigma and help them to understand the importance of supporting the adolescent and her child. *Supervisors* were the last cadre involved in the interventions; they mentored the Household Facilitators and Mentors with challenging cases and provided refresher trainings. The programme had 167 HVT members (48 HVTs) and each team worked with eight adolescents.

HVTs collaborated through a case management approach which included regular communication, care coordination and household follow‐up by a Mentor and Household Facilitator. The Mentor primarily focused on the adolescent client. The Household Facilitator focused on others in the household, especially males, and collaborated with local community‐based organisations to ensure adolescent participants were able to access services such as those for gender‐based violence (GBV) and livelihoods opportunities. Supervisors supported Mentor and Household Facilitator activities, including helping HVTs problem‐solve and effectively manage cases, which were particularly important due to the complex nature of many cases. This included helping HVTs respond and manage challenges such as the risk of self‐harm among adolescent participants and incidents of household abuse. Through at least two visits each month to the household by the Mentor (and depending on need, the Household Facilitator), HVTs ensured that adolescents received antenatal care (ANC), post‐natal care (PNC), HIV and other services; and that babies received necessary health and social services.

The JUA programme was implemented as a consortium with different roles and responsibilities for partners, acting as one team. Under USAID’s AIDSFree programme, led by John Snow, Inc. (JSI), in partnership with the Elizabeth Glaser Pediatric AIDS Foundation (EGPAF), four community‐based organisations led day‐to‐day implementation including recruitment and management of the HVTs listed as follows: Make Me Smile in Kisumu, Kagwa in Homa Bay, plus the Adventist Centre for Care and Support and St. John’s Community Centre in Nairobi. The AIDSFree team (JSI and EGPAF with the four community‐based organisations) designed the programme with active engagement of the communities, and County governments, in the respective counties throughout the design, implementation and monitoring processes.

Standard operating procedures and training materials were developed for use in implementation and in training the 167 HVT members – 80 individuals in January 2018 and 87 individuals in June 2018. A bi‐directional referral process was developed for use between HVT, health facilities, community‐based organisations, schools and other programmes, such as national insurance programmes and mental health services to facilitate linkages and networks. Up‐front discussions and meetings by the HVTs with management of relevant community health and social services ensured these facilities/staff were ready to offer pregnant adolescent girls and young mothers services (noting that adolescent clients had previously reported that community services would often turn them away and/or that they felt stigmatised). For any referral made, forms were issued in triplicate – one remaining in the HVT’s referral booklet, one given to the adolescent participant to share with the referee service delivery point and one returned to the HVT by the adolescent participant to ascertain completion of the referral.

### Adolescent participant selection

HVTs conducted an initial mapping, drawing upon existing registers and community networks to identify adolescent girls attending ANC and PNC. To prioritise participants for enrolment, we conducted comprehensive socioeconomic and health vulnerability screens to determine HIV status, school enrolment status, mental challenges, physical disabilities, household living arrangement and age. The screenings helped identify the most vulnerable adolescent girls in the community to be enrolled in the programme.

Assent from participants and consent from parents/guardians were obtained before screening for vulnerability. After acceptance for enrolment into the programme, a second phase of assent and consent was conducted with participants and parents/guardians, respectively. Both the consent and assent processes were offered as either written or oral, determined collaboratively with participants according to JSI and EGPAF protocols. Each enrolled client was given a unique study participant number. HIV status was not a criterion for enrolment into the programme; however, participants with unknown status were linked to HIV testing services upon enrolment.

### Ethical approvals

The AMREF Africa Ethics and Scientific Review Committee and the Advarra Institutional Review Board in the United States reviewed and approved the study protocol.

### Tool development

In addition to using existing validated tools, the programme developed a tool to measure household supportiveness (see Appendix S1). Indicators included family/caregiver’s: (i) participation in HVT visits; (ii) paying for services the adolescent was referred to; (iii) allowing the adolescent to remain living in the household throughout pregnancy and post‐partum period; (iv) providing follow‐up referral support; (v) supporting school retention/return; (vi) showing interest in the pregnancy and the infant’s development; (vii) supporting the adolescent’s ART adherence; and (viii) and helping to care for the infant.

### Data collection

Data collection took place from March 2018 to February 2019. Baseline, end line and routine data were collected on 25 indicators covering HIV, ANC and post‐natal care, family planning (FP), and post‐rape care at 36 health facilities for all participants enrolled in the programme at the time of data collection. Community‐based organisations reported data from client service registers on GBV, household economic strengthening, social protection and household reintegration (for girls who ran away/were sent away from home). Data collected from schools included registration and enrolment statistics. Data were also collected directly from participants during HVT visits and documented in programme case management data collection forms. Community‐based organisations leading each HVT were responsible for compiling data and reporting weekly progress and monthly data to the study team.

### Data management

Data were stored in the programme database hosted on a secure server at EGPAF’s Nairobi office. Using the unique study participant number, programme data staff merged the datasets from the different sources into a single de‐identified dataset, which was stored in a password‐protected Comma‐Separated Values (CSV) format file to enable import to other analytic programmes.

### Data analysis

Descriptive statistics comprising frequencies, averages and ranges, were used to summarise demographic, social and clinical characteristics of participants including age, gender, marital status and HIV status. Data were analysed using Microsoft Excel and Power BI, and the results disaggregated by month, age, client category (ANC or PNC) and other relevant characteristics. Two‐sample z tests were conducted to determine whether differences between baseline and endline measures for key indicators were statistically significant.

## Results

### Mother and infant characteristics

We enrolled 181 adolescent girls attending ANC and 203 girls attending PNC (Table 1). Nineteen participants had pre‐existing knowledge of their HIV‐positive status, and one individual was newly identified as HIV‐positive. Throughout implementation, 18 HIV‐positive participants remained active and two moved outside of the study areas. Only one participant had a known HIV‐positive infant at the time of enrolment. 93% of participants enrolled during pregnancy or post‐partum were 15‐19 years of age. 77% of those receiving PNC services enrolled with infants 6 months and younger.

**Table 1:** Characteristics of participants

Indicator	Pregnant	Post‐partum (0–24 months)	Total
Total enrolled	181 (47%)	203 (53%)	384
Known positives enrolled	6 (32%)	13 (68%)	19
Newly identified positive	1 (100%)	0 (0%)	1
Positives active at end of project	0 (0%)	18 (100%)	18
Known positive infants enrolled	0 (0%)	1 (100%)	1
Adolescent age at enrolment 10–14 years	11 (41%)	16 (59%)	27
Adolescent age at enrolment 15–19 years	159 (45%)	198 (55%)	357
Infant age at enrolment 0–6 months		270 (100%)	270
Infant age at enrolment 7–12 months		82 (100%)	82

By the programme end, 355 participants remained in the programme. Among those not retained, 3 were due to infant death, 1 due to miscarriage, 1 due to stillbirth, 1 because the adolescent matured beyond the beneficiary age requirement, and 23 moved out of the programme area.

### ART retention

**Figure 1:**
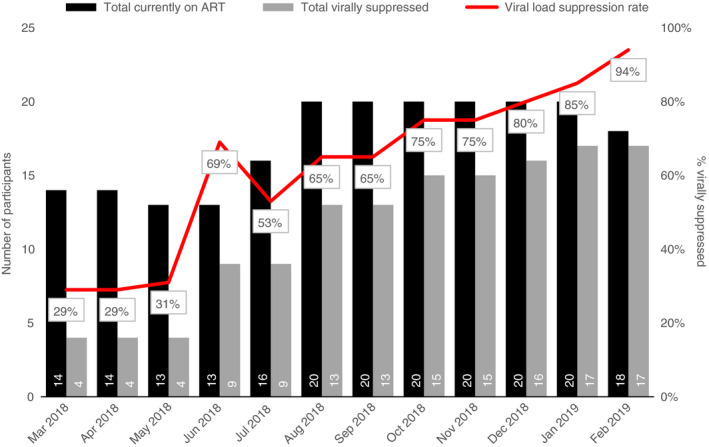
Viral suppression of HIV‐positive participants over time.

All HIV‐positive participants were initiated and retained on ART for six consecutive months of programme implementation (August 2018–January 2019). In Homa Bay, ten participants at baseline were HIV‐positive and on ART. Due to one infant death, only nine HIV‐positive participants continued throughout the life of the programme with 100% of them retained on ART by the final month of measurement (February 2019). In Kisumu and Nairobi counties, 100% of participants were retained on ART (Figure 1).

### Viral suppression of HIV‐positive participants

The viral suppression rate for HIV‐positive adolescent participants was 29% across all study sites (Figure 1), which increased to 97% by programme end (*P* < 0.0001), with 94% of those in Homa Bay and 100% of those in Kisumu and Nairobi being virally suppressed by the end of the intervention.

### Mother‐to‐child transmission

Among the 17 HIV‐exposed infants at the end of the programme, 100% had an up‐to‐date PCR test at each of the required ages (6 weeks, 6 months and 12 months per Kenya PMTCT guidelines), and none seroconverted during programme implementation.

### Skilled birth attendance

Among the 181 ANC participants, 178 had given birth by programme end, with 168 (94%) being assisted by a skilled birth attendant during delivery, which was significantly higher than the 78% of PNC participants who had delivered under such care at the time of enrolment (*P* < 0.001). Skilled birth attendance was 96% in Nairobi, 98% in Homa Bay and 89% in Kisumu County by programme end.

### FP uptake

**Figure 2:**
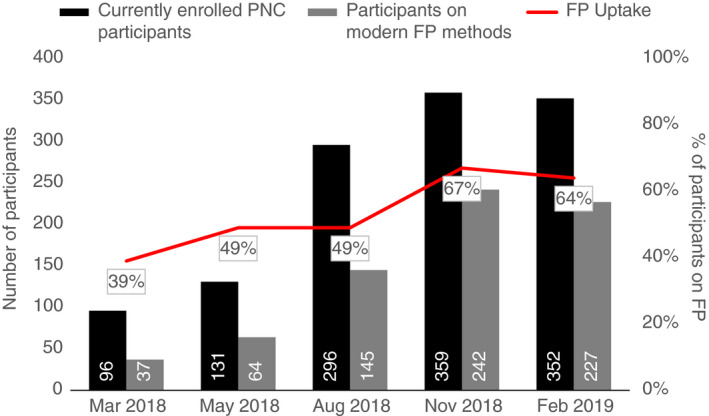
Modern FP method use among participants.

Modern FP method use at baseline was 39% (Figure 2) which increased to 67% by November 2018 as more participants gave birth, before plateauing at 64% in February 2019 (*P* < 0.001). Homa Bay County had the greatest increase in uptake, from 28% to 79%, representing a 51 percentage‐point change. Uptake in Kisumu increased from 52% to 81% between March 2018 and January 2019, however, by February 2019, use had declined to 57%. In Nairobi, uptake increased from 16% to 60%.

### Referrals to health and social services

Referrals ranged from 52 to 275 per month with completion rates ranging between 61% and 92% per month. The average referral completion rate was 80% with no discernible differences in the likelihood of referral completion by month. Referral completion was 84% at baseline and 78% at end line. Referrals were most often made for PNC, HIV and other services including clinical care for infants when they became sick, clinical reviews for adolescents and civil registration. Participants most often completed referrals for GBV, educational or economic support services. Referrals made for mental health/psychosocial services were infrequently completed (Table 2).

**Table 2:** Referral completion rate by service type

Type of referral	Referrals made	Referrals completed	Completion rate
Antenatal care services	162	122	75.3%
Postnatal care services	276	209	75.7%
Gender‐based violence services	29	28	96.5%
Human immunodeficiency virus services	280	240	85.7%
Psychosocial/mental health services	46	8	17.3%
Educational services	91	87	95.6%
Economic support services	29	28	96.5%
Other referrals	426	330	77.4%
Total	1339	1052	78.5%

### Household supportiveness

At baseline, 56 (14%) of the 384 beneficiaries were from non‐supportive households; 111 (29%) were from partially supportive households; and 217 (57%) were from fully supportive households. At end line, 100% of the 56 baseline non‐supportive households had transitioned to being partially or fully supportive due to efforts of HVT. Among the 355 active beneficiaries at end line, 303 (85%) were from fully supportive households and 52 (15%) from partially supportive households. Over the life of the programme, household support increased from 57% to 85%.

### School re‐admission

Among all 355 participants eligible for school, only 25% were in school at the programme end. The remaining 75% of participants were involved in caring for their infants during the post‐partum period.

## Discussion

This paper describes one of the first home visiting programmes tailored for pregnant adolescent girls, adolescent mothers and their infants in a high HIV prevalence setting in sub‐Saharan Africa. The model was designed in close collaboration with community members, ensuring the design reflected the needs of adolescents and received support from the community from the onset. The model was unique as it engaged peer mentors and household facilitators – both male and female – to work with caregivers, partners and other family and community members to provide an enabling environment for improving the health and social outcomes of adolescent mothers and their infants. Supervisors ensured the HVT team was well‐supported and that there were quality assurance processes in place. The model strengthened the network of care providers, including ensuring bi‐directional referrals between HVTs, community‐based organisations, health facilities, schools and other services. Further, the programme built the case management skills of HVTs not only to incorporate routine health and social assessments into home visits, but also to make educated and independent care decisions based on the unique needs of participants.

Results showed marked improvements in multiple indicators. By the end of the programme, 94% of participants delivered with a skilled birth attendant, which is higher than the averages of 91% for Nairobi and 71.9% for Kisumu counties, respectively, based on the 2014 Kenya Demographic and Health Survey [2]. It was also higher than the level of skilled birth attendance (78%) among PNC participants enrolled in the programme. The high levels of skilled birth attendance may be a result of continuous education on the benefits of skilled birth provided by the HVTs, and linkages to national insurance, which covers the cost of skilled deliveries.

There was a significant increase in the proportion of HIV‐positive adolescent mothers whose viral load was suppressed, from about a third at baseline to all adolescent mothers at end line, which was higher than the national average of 65% [26]. In addition, the proportion of HIV‐positive adolescent mothers retained in care and on ART was consistent with the global target of 90%. There was no transmission of HIV among ANC participants in the study, unlike the national average of 12% [22]. Programme components that likely contributed to the high viral suppression, retention in care and use of PMTCT services included home‐based education for the adolescent girls and their family members on the importance of ART prophylaxis, adherence and retention. Escorted referrals to health and social services during programme initiation, and home‐based follow‐up for participants who did not honour appointments to resolve non‐attendance issues, also likely played a strong role.

In Kenya, 19% of sexually active girls aged 15–17 years and 28% of sexually active girls aged 18–19 years use modern contraception, indicating that significant coverage gaps remain, particularly for younger adolescents [27]. Increased uptake of modern contraception during the post‐partum period by programme end may be attributed to home‐based antenatal and post‐partum education regarding FP and linkages to post‐partum FP services. The small decline in FP use may in part be attributed to participants who were on long‐term contraceptive methods and did not need to renew during the study period (and due to programme ending, HVT were unable to track referral completion).

The bi‐directional referral process and tools developed by the programme for use between the participating health facilities and community‐based organisations enabled participants to more easily access services. Despite these efforts, the referral completion rate declined from 84% at baseline to 78% at end line. This may be because the escorted referral process offered at baseline declined over time, as by programme end, emphasis was on increasing assertiveness of participants and empowering them regarding their own health, including referral uptake. The low uptake of mental health/psychosocial service referrals (17.3%) may be due to stigma associated with seeking mental health services; and/or the limited availability of these services specific to adolescent’s needs. Given evidence on the prevalence of mental ill‐health among pregnant and post‐partum adolescent girls, efforts to provide these services in future programmes is essential [28, 29, 30, 31].

Kenya’s national school readmission policy states that a pregnant school‐going adolescent girl is to continue with her education until the time she delivers and school‐going mothers should be allowed back to school after childbirth [32]. The majority (75%) of participants did not re‐enter in school demonstrating the importance of integrating innovative models to improve attendance among adolescent mothers, including removing school fees and targeted financial support/cash transfers, allowing time outside of class to breastfeed and attend PNC appointments and supporting childcare/placing day cares in schools [33, 34]. Such interventions require advocacy for policy changes, school administrative support and training for school counsellors and other staff to make the school environment flexible and welcoming for adolescent mothers [5, 35].

Some of the most promising findings of this study pertain to changes in household supportiveness, which were evident in positive changes in attitudes of family members. Household members play an important role in the lives of their adolescent girls, influencing girls’ ability to receive medical care, attend school and work outside the home [36, 37]. The engagement of household members during visits/counselling sessions enhanced the relationship between them and the adolescent girls. Typically, the male HVT member worked closely with men and boys in the lives of adolescent girls to foster their support for the adolescent mother and her baby, including how best to support the pregnancy and counselling on safer sex practices for male partners. Examples of increased supportiveness included parents who were initially reluctant to allow their daughters to attend ANC appointments, return to school, and use post‐partum FP, and later provided financial support to attend ANC appointments and school and offered childcare so that adolescent mothers could attend school. Additionally, male siblings who were initially hostile towards pregnant adolescent girls then became engaged contributors to the child’s care. Household supportiveness was largely attributed to HVTs mentoring adolescent girls on how to effectively communicate with household heads, and HVT sensitisation of household members and others to reduce barriers to health‐seeking behaviours, address stigma and optimise support for adolescents and their babies.

The findings of this study may be influenced by certain limitations. The experiences of the 384 purposively selected participants may not necessarily reflect those of all adolescent mothers in the study counties. Additionally, the sample size was not powered to detect statistically significant changes in indicators of interest over time. Data analysis did not control for confounding factors to allow attributing changes in the indicators of interest to programme effect. There may also be competing explanations that may contribute to the observed changes such as support from other initiatives.

There could also be questions about scalability and sustainability of the programme. Further costing studies, as well as secondary impact studies of the programme on household members, HVT members and the broader community are important for assessing costs and impact to inform scale‐up or replication. Potential cost‐savings for the programme may include integrating the key curricula and processes into existing community‐based programming and conducting more group‐based case management sessions instead of one‐to‐one sessions with adolescent girls.

In spite of the limitations, the findings of this study provide evidence that an innovative home visiting programme for HIV‐vulnerable pregnant and post‐partum adolescent girls has potential to significantly improve ART retention, viral suppression, mother‐to‐child transmission, skilled birth attendance and FP uptake. The case‐management, team‐oriented model of working with not only the adolescent girl herself, but also her parents and community members, as well as engaging men, helped address critical socio‐cultural and behavioural barriers to accessing and utilising services. Further research on cost‐effective, promising elements of such home visiting models for improving health and social outcomes for pregnant adolescent girls, adolescent mothers and their children is essential.

## Supporting information


**Appendix S1.** Household Supportiveness Assessment Form.

**Appendix S2.** Indicator List.
