# Supplementary material for: Health and social outcomes of HIV‐vulnerable and HIV‐positive pregnant and post‐partum adolescents and infants enrolled in a home visiting team programme in Kenya
Source: Trop Med Int Health. 2021 Mar 25;26(6):640–8. doi: 10.1111/tmi.13568 (PMC9291167; doi:10.1111/tmi.13568)
Supplement: Supplementary file 1 — Appendix S1. Household Supportiveness Assessment Form. [file TMI-26-640-s002.docx]

**Supplement 1: Household Supportiveness Assessment Form**

Client Name: _____________ ___ Age: Date: _____________

|  | **HOUSEHOLD ASSESSMENT FORM** | | |
| --- | --- | --- | --- |
|  | **Questions** | **Responses** | **Scoring criteria** |
| 1 | Is parent(s)/ caregiver/ partner willing to participate/attend household visit | - Yes - No   If yes, how often   - Once a month - Twice a month - Always | Assign 3 points if answer is yes. |
| 2 | Does the parent(s) /caregiver/ partner agree to pay for services the adolescent has been referred for? | - Yes - No | Assign 2 points if answer is yes |
| 3 | Did the parent(s) /caregiver/ partner allow adolescent to stay within the household during pregnancy and/or lactation | - Yes - No | Assign 2 points if answer is yes |
| 4 | Does the parent(s) /caregiver/ partner make follow-ups on referrals made | - Yes - No | Assign 2 points if answer is yes |
| 5 | Does the parent(s) /caregiver/ partner show interest in the pregnancy and infant milestones | - Yes - No | Assign 3 points if answer is yes |
| 6 | Is the family in support of readmission to school for the adolescent | - Yes - No | Assign 2 points if answer is yes |
| 7 | Does the parent(s) /caregiver/ partner support the adolescents with adherence  *(For HIV positive adolescent)* | - Yes - No | Assign 1 points if answer is yes |
| 8 | Does the household accept to support in caring for the infant take care of infant when adolescent is not in  *(For households with PNC beneficiaries)* | - Yes - No | Assign 1 points if answer is yes |

| **Notes:**  For ANC, household supportiveness level is based on sum of scores from number 1-6.  For PNC, household supportiveness level is based on sum of scores from number 1-6 and 8 | |
| --- | --- |
| **Supportiveness level:** ANC  Fully supportive – If score is between 11 – 14  Partially supportive – If score is between 6 – 10  Not supportive – If score is between 0 - 5 | **Supportiveness level:** PNC  Fully supportive – If score is between 12 – 15  Partially supportive – If score is between 7 – 11  Not supportive – If score is between 0 – 6 |

Name on Interviewer: _______________________________________________________
