# Supplementary material for: Health and social outcomes of HIV‐vulnerable and HIV‐positive pregnant and post‐partum adolescents and infants enrolled in a home visiting team programme in Kenya
Source: Trop Med Int Health. 2021 Mar 25;26(6):640–8. doi: 10.1111/tmi.13568 (PMC9291167; doi:10.1111/tmi.13568)
Supplement: Supplementary file 2 — Appendix S2. Indicator List. [file TMI-26-640-s001.docx]

**Supplement 2: Indicator List**

# of household facilitators, mentors and supervisors trained

# of newly identified pregnant adolescent girls / adolescent mothers and their infants enrolled into the home visiting program (disaggregated by district and two year age bands)

# of pregnant adolescents/adolescent mothers enrolled in Cash Plus Care program (disaggregated by district and two year age bands)

# of newly identified pregnant adolescent girls / adolescent mothers and their infants by the home visiting program (HVP)

# of newly identified pregnant adolescent girls / adolescent mothers and their infants enrolled into the home visiting program (HVP)

# of adolescent mothers on a modern family planning method

# of pregnant adolescent girls / adolescent mothers receiving the prescribed minimum number of home visits during pregnancy

# of pregnant adolescents / adolescent mothers enrolled in Condition Cash Transfer program

# of referrals made to health and social protection services

# of the priority populations reached with the standardized, evidence-based intervention(s) required that are designed to promote the adoption of HIV prevention behaviors and service uptake

# of individuals who received HIV Testing Services (HTS) and received their test results, disaggregated by HIV result

% of pregnant adolescents (10-19) with known HIV status at antenatal care (includes those who already knew their HIV status prior to ANC), disaggregated by HIV result

# of adolescents and children newly enrolled on antiretroviral therapy (ART)

# of adolescent girls, their partners and children currently receiving antiretroviral therapy (ART)

% of HIV-positive pregnant adolescents who received ART to reduce the risk of mother-to-child-transmission (MTCT) during pregnancy

# of infants born to HIV infected adolescent mothers who had a virologic HIV test within 12 months of birth during the reporting period

# of infants born to HIV infected adolescent mothers who had an up to date appropriate immunization

# of adolescent mother-infants pairs keeping post-natal/Child Welfare Services appointments

# of infants born to adolescent mothers who have age appropriate developmental milestones

# of pregnant adolescent/mothers with completed referrals for social protection

# of pregnant adolescent girls/adolescent mothers receiving post-GBV care

# of adolescent mother re-admitted to school after childbirth

# of pregnant adolescents retained in school during pregnancy

# of households supporting the pregnant adolescent to access Health and social services

% of enrolled girls and young mothers retained in the program at 12 months after enrollment
